# Accurate Prediction of Inhibitor Binding to HIV-1 Protease Using CANDOCK

**DOI:** 10.3389/fchem.2021.775513

**Published:** 2022-01-17

**Authors:** Zackary Falls, Jonathan Fine, Gaurav Chopra, Ram Samudrala

**Affiliations:** ^1^ Department of Biomedical Informatics, Jacobs School of Medicine and Biomedical Sciences, University at Buffalo, State University of New York, Buffalo, NY, United States; ^2^ Department of Chemistry, Purdue University, West Lafayette, IN, United States; ^3^ Purdue Institute for Drug Discovery, West Lafayette, IN, United States; ^4^ Purdue Center for Cancer Research, West Lafayette, IN, United States; ^5^ Purdue Institute for Inflammation, Immunology and Infectious Disease, West Lafayette, IN, United States; ^6^ Purdue Institute for Integrative Neuroscience, West Lafayette, IN, United States

**Keywords:** molecular docking, inhibitor prediction, protein–ligand interaction, HIV-1 protease, knowledge-based force field, CANDOCK

## Abstract

The human immunodeficiency virus 1 (HIV-1) protease is an important target for treating HIV infection. Our goal was to benchmark a novel molecular docking protocol and determine its effectiveness as a therapeutic repurposing tool by predicting inhibitor potency to this target. To accomplish this, we predicted the relative binding scores of various inhibitors of the protease using CANDOCK, a hierarchical fragment-based docking protocol with a knowledge-based scoring function. We first used a set of 30 HIV-1 protease complexes as an initial benchmark to optimize the parameters for CANDOCK. We then compared the results from CANDOCK to two other popular molecular docking protocols Autodock Vina and Smina. Our results showed that CANDOCK is superior to both of these protocols in terms of correlating predicted binding scores to experimental binding affinities with a Pearson coefficient of 0.62 compared to 0.48 and 0.49 for Vina and Smina, respectively. We further leveraged the Database of Useful Decoys: Enhanced (DUD-E) HIV protease set to ascertain the effectiveness of each protocol in discriminating active versus decoy ligands for proteases. CANDOCK again displayed better efficacy over the other commonly used molecular docking protocols with area under the receiver operating characteristic curve (AUROC) of 0.94 compared to 0.71 and 0.74 for Vina and Smina. These findings support the utility of CANDOCK to help discover novel therapeutics that effectively inhibit HIV-1 and possibly other retroviral proteases.

## 1 Introduction

Inhibition of the Human immunodeficiency virus (HIV) protease blocks viral maturation and replication, making inhibitors of this vital enzyme an important class of drugs for the treatment of HIV infection (Wlodawer et al., 1989; Wlodawer and Vondrasek, 1998). The introduction of HIV protease inhibitors reduced the mortality rate of infected patients in the US significantly, from about 50 thousand deaths per year in 1995, down to 20 thousand by 2000 (Centers for Disease Control and Prevention, 2001a; Centers for Disease Control and Prevention, 2001b; Quinn, 2008). However, escape mutations within the viral protease have resulted in HIV strains that are resistant to these inhibitors, presenting a challenge to identify which protease inhibitors are effective against specific mutants or discover and design broad spectrum inhibitors (Ohtaka and Freire, 2005). For these reasons, accurate prediction of inhibitor efficacy against protease mutants that arise during infection has been a focus of HIV drug discovery for decades.

Previous efforts to predict inhibitor activity against human immunodeficiency virus 1 (HIV-1) proteases include rule-based methods (Shafer et al., 1999; Kantor et al., 2001), support vector machine (SVM) models (Beerenwinkel et al., 2002; Cai et al., 2003), chemical shape and features (Yadav et al., 2012; Pandit et al., 2006; DesJarlais and Dixon, 1994; Wei et al., 2015), various docking protocols (Chang et al., 2007, 2010), and molecular dynamics (MD) simulations (Rick et al., 1998; Wang and Kollman, 2001; Wang K. et al., 2004; Jenwitheesuk and Samudrala, 2003; Jenwitheesuk et al., 2004; Jenwitheesuk and Samudrala, 2005a; Jenwitheesuk and Samudrala, 2005b). These different approaches have displayed varying results. Chang et al*.* compared the efficacy of Autodock 4 and Autodock Vina in predicting active versus inactive compounds using the National Cancer Institute Diversity II compound sets and showed that both protocols perform better than random [area under the curve (AUC) of 0.69 and 0.68, respectively] on this diverse compound set (Huang et al., 2006; Chang et al., 2010). Pandit et al. generated a pharmacophore model using Molecular Operating Environment (MOE) software to predict activity from a set of known protease inhibitors and non-inhibitors, correctly identifying 65 of the 75 protease inhibitors and incorrectly classifying 11 out of 75 non-inhibitors. When volume exclusion was incorporated into the model, the authors were able to decrease the number of false positives to 5 out of 75 while reducing the true positives to 60 out of 75. (Pandit et al., 2006). These results display the tradeoff between sensitivity and specificity and the limitations of this model.

The problem is more difficult in a *de novo* molecular docking scenario, when the x-ray diffraction structure of a protease mutant (or homology model) is docked to an inhibitor and the necessary rotations and transformations are calculated without *a priori* knowledge (Razzaghi-Asl et al., 2015). In contrast, approaches that use x-ray diffraction structure poses for the protease and inhibitor, combined with the features of the compound and/or MD, to predict binding affinities have met with some success (Jenwitheesuk et al., 2004; Jenwitheesuk and Samudrala, 2005a; Jenwitheesuk and Samudrala, 2005b). Jenwitheesuk and Samudrala obtained a peak correlation of 0.87 between predicted binding energies and experimental binding affinities through the use of MD simulations (Jenwitheesuk and Samudrala, 2003). These MD approaches have the advantages of allowing for the exploration of the inhibitor within the binding site and using scoring functions that are sensitive to amino acid mutations in the protease structure. However, MD requires an accurate 3D complex structure of the bound pose between ligand and protein as a starting point, thus limiting this method by requiring solved or modeled structure(s) and/or a docking protocol. Jenwitheesuk and Samudrala showed that calculated versus experimental binding correlation was 0.38 with the docking protocol Autodock alone, illustrating the benefit MD provides (Jenwitheesuk and Samudrala, 2003). Leveraging docking protocols with more sophisticated and robust scoring functions can be used to identify the pose of the inhibitors with respect to the protease as well as accurately predict the corresponding binding scores.

Here we used a hierarchical fragment-based docking based dynamics protocol implemented in the CANDOCK protocol (Fine and Chopra, 2018; Fine et al., 2020), in conjunction with its all-atom knowledge-based scoring function (Bernard and Samudrala, 2009), to predict the binding scores of inhibitors to HIV-1 protease. The knowledge-based scoring function calculates and optimizes atomic interactions in the binding pocket to sample biologically relevant ligand conformations giving us an ability to identify specific interactions (beyond hydrogen bonding or pi-stacking, etc.) in protein binding sites. We first optimized the parameters for the knowledge-based scoring function used in CANDOCK using a set of HIV-1 protease–inhibitor complex structures with known binding affinities from BindingMOAD (Smith et al., 2019). We then used CANDOCK to predict actives versus decoys from the Directory of Useful Decoys Enhanced (DUD-E) HIV protease subset (Mysinger et al., 2012) to affirm the discriminatory abilities of the improved protocol and compared it to two popular molecular docking methods, AutoDock Vina and Smina. Our research showed a strong correlation between experimental binding affinities and predicted binding scores for CANDOCK which resulted in a 0.62 Pearson coefficient compared to the 0.48 and 0.49 for Vina and Smina, respectively. In addition, the performance of CANDOCK on the DUD-E HIV protease set exceeded Vina and Smina with an area under the receiver operating characteristic curve (AUROC) of 0.94 for CANDOCK compared to 0.71 for Vina and 0.74 for Smina. These results demonstrate the predictive power of CANDOCK for the specific case of assessing inhibitor potency against HIV-1 protease.

## 2 Materials and Methods

### 2.1 Curation of Human Immunodeficiency Virus 1 Protease–Inhibitor Sets for Benchmarking

We compiled a set of 30 HIV-1 protease–inhibitor complex structures (Table 1) extracted from the Protein Data Bank (PDB), all of which were solved using x-ray diffraction with a resolution of 2.5 Å or lower and have experimentally determined binding affinities between the protease and given inhibitor (Berman et al., 2000). To use these structures in our docking simulations, we had to separate and process the protease and inhibitor in each complex. First, the proteases were processed using biopython and OpenMM to remove any co-crystallized ligands so the HIV-1 protease dimer was all that remained to be used as the receptor in each molecular docking simulation (Cock et al., 2009; Eastman et al., 2017). The inhibitor was also separated from the corresponding complex in each case and converted to a Mol2 file format using OpenBabel for compatibility with the CANDOCK protocol (O'Boyle et al., 2011, 2008). Both the protease and inhibitor files for each complex were also converted to the PDBQT file format using AutoDocktools for compatibility with the AutoDock Vina protocol (Morris et al., 2009). The binding sites for each protease were defined using the coordinates of the native ligand in the PDB structure. Experimentally observed inhibition constants, K_*i*_, for the bound ligands in all 30 complexes were obtained from BindingMOAD (Smith et al., 2019).

**TABLE 1:** List of HIV-1 protease structures extracted from Protein DataBase (PDB) with experimentally determined inhibition constants **(** **K**_***i***_ **)** extracted from BindingMOAD.

PDB ID	Resolution (Å)	Ligand ID	Experimental K_*i*_ (M)
1a8g	2.50	2Z4	7.40e−09
1aaq	2.50	PSI	3.00e−09
1aid	2.20	THK	1.50e−05
1ajv	2.00	NMB	1.91e−08
1ajx	2.00	AH1	1.22e−08
1g2k	1.95	NM1	1.10e−08
1g35	1.80	AHF	7.30e−09
1gno	2.30	U0E	2.00e−08
1hbv	2.30	GAN	4.30e−07
1heg	2.20	PSI	1.80e−08
1hih	2.20	C20	9.00e−09
1hiv	2.00	1ZK	1.00e−09
1hos	2.30	PHP	2.80e−09
1hps	2.30	RUN	6.00e−10
1hpv	1.90	478	6.00e−10
1hpx	2.00	KNI	5.50e−12
1hvh	1.80	Q82	1.10e−08
1hvi	1.80	A77	8.40e−11
1hvj	2.00	A78	4.00e−12
1hvk	1.80	A79	7.70e−11
1hvl	1.80	A76	1.00e−09
1hvr	1.80	XK2	3.10e−10
1hvs	2.25	A77	5.00e−11
1pro	1.80	A88	5.00e−12
1qbr	1.80	XV6	2.70e−11
1qbs	1.80	DMP	3.40e−10
1qbt	2.10	146	2.40e−11
1qbu	1.80	846	5.80e−11
1sbg	2.30	IM1	1.80e−08
1sdt	1.30	MK1	5.40e−10

The PDB ID and resolution, in angstroms, are provided as well as the corresponding co-crystallized inhibitor ligand ID and inhibition constant (M) for the protease–inhibitor complex.

In addition to the 30 HIV-1 protease–inhibitor complexes, we also extracted a set of active and decoy compounds for the HIV protease from the DUD-E which consisted of 1,395 actives and 36,278 decoys against the macromolecule dimer (Mysinger et al., 2012).

Lastly, a set of 14 compounds were extracted from PubChem, seven of which were experimentally determined to be active HIV-1 protease inhibitors and the other seven were determined to be inactive compounds. The list of compounds and their corresponding activity against HIV-1 protease is given in Table 2.

**TABLE 2:** Compounds extracted from PubChem with experimentally determined inhibition activity against HIV-1 protease.

PubChem compound ID	HIV-1 protease activity
CID480440	Active
CID480447	Active
CID480550	Active
CID514961	Active
CID480441	Active
CID480469	Active
CID514958	Active
CID10509626	Inactive
CID478338	Inactive
CID49796249	Inactive
CID66162	Inactive
CID10747313	Inactive
CID478339	Inactive
CID49796254	Inactive

The PubChem compound identifier and the corresponding activity is provided for each of the 14 compounds.

### 2.2 Molecular Docking Protocols

Three different molecular docking protocols: CANDOCK (Fine and Chopra, 2018; Fine et al., 2020), Autodock Vina (Trott and Olson, 2010), and Smina (Koes et al., 2013) were used herein to predict the binding affinity between the HIV-1 protease inhibitors and the protease macromolecule binding sites.

#### 2.2.1 CANDOCK

CANDOCK is a hierarchical fragment-based docking with dynamics protocol to “grow” the ligand in the binding pocket by leveraging a generalized knowledge-based statistical scoring function to identify docked poses (Bernard and Samudrala, 2009; Fine and Chopra, 2018). The fragment-based approach identifies and breaks rotatable bonds in the ligand to reduce the ligand down to rigid subunits, which are then individually docked in the binding pocket and relinked to build the complete original ligand in the best pose. This method enables comprehensive search of both the binding pocket and the conformational space of ligand. The scoring parameters for the knowledge-based scoring function were varied during this study and are discussed in Section 2.3. The protease–inhibitor binding site was defined as a sum of centroids, with 4.5 Å radii, at each atom of the known bound ligand for each complex. We also used the Generalized Amber ForceField (GAFF) implemented in CANDOCK as a control for the knowledge-based scoring function.

#### 2.2.2 AutoDock Vina

AutoDock Vina, referred to here as Vina, is a well-known molecular docking protocol that uses a physics-based forcefield, similar to X-Score, that is tuned on the experimental data in PDBBind (Wang et al., 2002; Trott and Olson, 2010; Su et al., 2018). In this study, Vina was used with default parameters with the following exceptions: the exhaustiveness and num_modes parameters set to 8 and 9, respectively. The binding box center was placed at the geometric center of the known bound ligand for each corresponding crystal structure. The length of all sides of the binding box were defined as two times the radius of gyration of the compound plus 9.0 Å, to ensure a large enough search space while simultaneously mimicking the binding site centroids used in CANDOCK.

#### 2.2.3 Smina

Smina is a forked version of Vina with expanded functionality and enabled user-defined scoring functions (Koes et al., 2013). In addition, Koes et al. modified the existing Vina potential by optimizing additional energetic terms found in the original code that were not used; i.e., the coefficients were set to 0. The potential terms included an electrostatic term, a desolvation term, and a non-hydrophobic contact term, among others that were all parameterized by training on the CSAR (Community Structure-Activity Resource) 2010 dataset (Dunbar Jr et al., 2011). The parameters used for Vina, described above, were also used here. The binding box was also defined identically.

### 2.3 Selector and Ranker

The CANDOCK protocol generates hundreds to thousands of binding modes for a protein–ligand pair that may then be scored. To obtain a single score, the protocol orders all of these modes by score using a knowledge-based forcefield (KBF) and chooses the top result as the best binding pose and corresponding binding score. We refer to this scoring and sorting as the *selector*. Once the top binding pose is chosen, we rescore this pose using the same or different variation of the KBF, by modifying one or more of the terms. The new score is then used in the calculation of the correlation between known and predicted binding affinities. We call the parameter set used for rescoring the *ranker* (Fine et al., 2020).

The KBF is an atomic level forcefield that is generalized to all intermolecular binding interactions, e.g., protein–small molecule, protein–DNA, etc. (Bernard and Samudrala, 2009). The equation to calculate the interaction score between two molecules, Eq. 1, is analogous to the net potential of mean force:

$$S\left(\left\{r_{ab}^{ij}\right\}\right)=-\sum \ln\frac{P\left(\overset{ij}{\underset{ab}{r}}|C\right)}{P\left(r^{ij}\right)}$$ (1)

This equation calculates the probability of two intermolecular atoms being within a given distance $\left(P\left(r_{ab}^{ij}|C\right)\right)$ with respect to the probability of any two atoms being within the same distance (*P*(*r* ^*ij*^)), where $r_{ab}^{ij}$ is the distance between atom *i* of type *a* and *j* of type *b*. For this function the distances, *r*, are discretized into distinct spherical shells. The KBF has four terms that affect the function and are optimized herein. The four terms are *functional*, *reference*, *composition*, and *cutoff*.

The probability distributions $\left(\right.P\left(r_{ab}^{ij}|C\right)$ and *P*(*r* ^*ij*^) are generated by one of two *functionals*: a normalized frequency distribution function (f), or a radial distribution function (r). The former assesses how many atoms are within a given shell, whereas the latter divides the number of atoms in the shell by the volume of the spherical shell.

The *reference* probability is calculated one of two ways: cumulative (*c*) or mean (*m*), which are the sum over all atom pairs or averaged over the number of atom pairs, respectively.

The last two terms, *composition* and *cutoff*, refer to the atom type pairs and the maximum distance (*r*) considered for calculations. The two options for *composition* are complete (c), which enables the use of all atom type pairs, and reduced (r), which limits the summation to only atom types *a* and *b* found within the given intermolecular complex. *Cutoff* can range from 4 to 15 Å.

The probabilities $\left(\right.P\left(r_{ab}^{ij}|C\right)$ and *P*(*r* ^*ij*^) were generated using the experimental data found in the Cambridge Structural Database (CSD) for both the protein–ligand and protein–DNA interactions (Allen, 2002).

The “default” parameter set for CANDOCK was defined as rmr6–rmr6, which equates to radial *functional*, mean *reference*, reduced *composition*, and 6 Å *cutoff* for both the selector and ranker.

## 3 Results and Discussion

### 3.1 Comprehensive Analysis of Parameters for the CANDOCK Knowledge-Based Scoring Function

The CANDOCK protocol uses a generalized statistical scoring function for scoring molecular interactions. This KBF is implemented with four different parameters: *functional*, *reference*, *composition*, and *cutoff* (Section 2.3). Each of the first three parameters have two options, and cutoff can range from 4 to 15 Å, resulting in 96 variations of the KBF. We generated the top poses for each of the 30 HIV-1 protease–inhibitor complexes using the 96 KBF variations for both the selector and ranker to ascertain the optimal combination.

**FIGURE 1:**
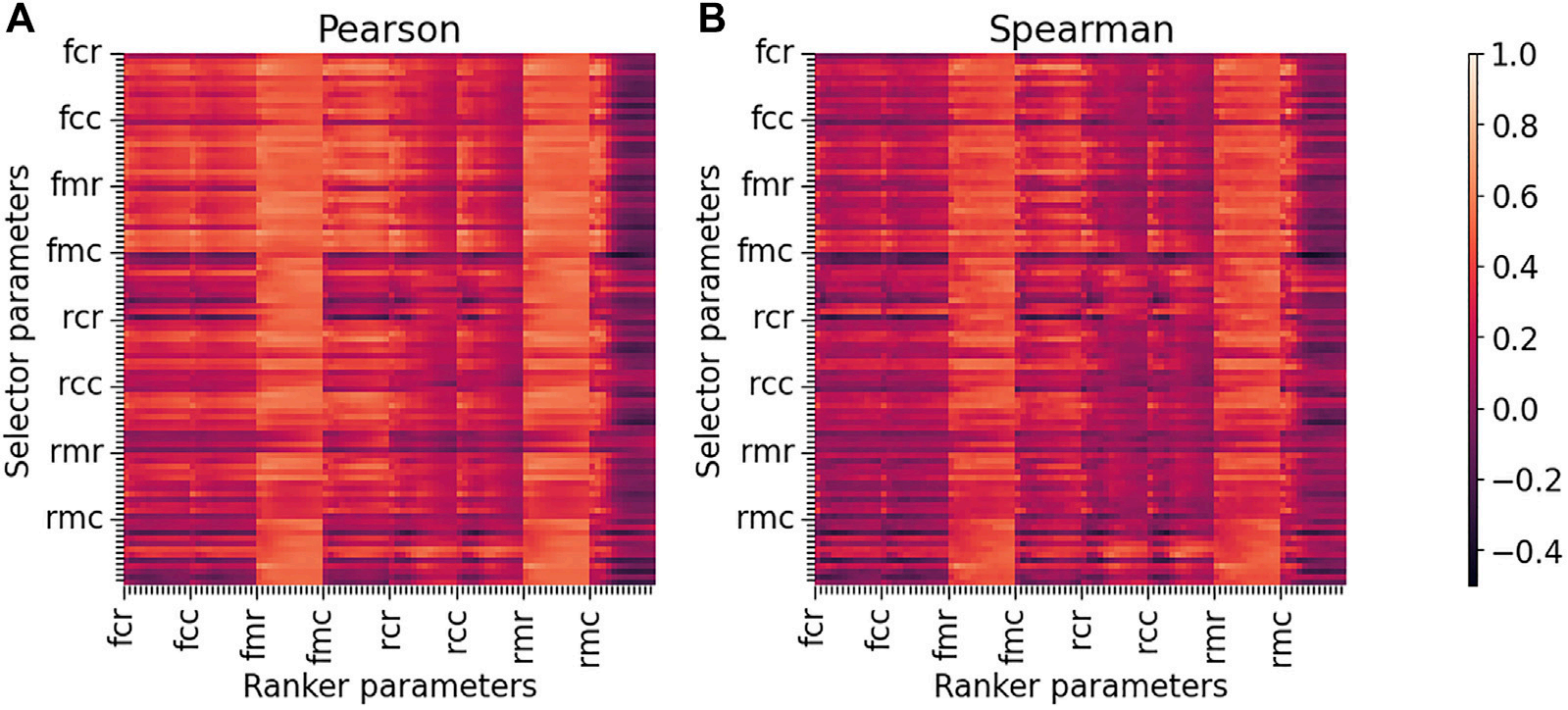
Heatmaps of Pearson and Spearman correlations between predicted binding scores and experimental binding affinities for all selector and ranker parameter sets in CANDOCK. The Pearson **(A)** and Spearman **(B)** correlations were calculated using 30 HIV-1 protease–inhibitor complexes where each top pose was scored using a specific selector and then rescored using a specific ranker. This analysis was performed for all 96 variations of each ranker/selector pair resulting in 9,216 correlations plotted in each heatmap—lighter orange color indicating stronger positive correlation. The *functional*, *reference*, and *composition* parameters are denoted on the major ticks of the horizontal and vertical axes. The minor ticks for each parameter set account for the 12 different *cutoffs* that can be used (4–15 Å). For both the Pearson and Spearman heatmaps, fmr and rmr rankers result in high correlations regardless of the selector used, demonstrating that the ranker chosen is more important than the selector used. The heatmaps also show that the rmr parameter set, which is the default for CANDOCK, is a strong performing ranker.

We calculated top poses and corresponding binding scores for the 30 HIV-1 protease–inhibitor complexes using all 96 variations of the KBF (selector) and subsequently rescored the top pose for all complexes with each of the 96 KBF variations (ranker), resulting in 9,216 different selector–ranker combinations. For each selector–ranker pair, we calculated the Pearson and Spearman correlation between the known K_*i*_ from BindingMOAD and the predicted binding scores for all 30 protease–inhibitor pairs. The correlations calculated showed us how accurately each selector–ranker parameter set predicted binding scores when compared to the experimental binding affinities. We populated a heatmap with all of the calculated correlations presented in Figure 1. This visualization showed us that rmr and fmr with all cutoffs were very high-performing rankers, where variation of the selector shows negligible effect on the correlations. These results were interesting from a high-level perspective by showing the efficacy of the fmr and rmr rankers, but we wanted to assess specific parameter sets to determine which would be the most accurate for the HIV-1 protease–inhibitor complexes.

The CANDOCK protocol was previously parameterized on the complete CASF-2016, containing 285 protein–ligand complexes across 57 proteins (Fine and Chopra, 2018; Su et al., 2018; Fine et al., 2020). Results from this analysis determined rmr6 as selector and rmc15 as ranker were the best performing parameter sets, and varying the selector did not have a major impact on the resulting correlations between binding affinity and binding score. The authors also commented on the results for the subset of HIV protease–inhibitor complexes in CASF-2016 stating that rmc15–rmr6, the reverse of the previously stated selector–ranker, was the best performing set. We used the knowledge obtained in this earlier study along with our own analysis of the heatmap of all correlations (Figure 1) to justify rmr6–rmr6 as our “default” selector–ranker because it is the default values set in the CANDOCK program, the rmr6 ranker was previously shown to be the best for the HIV protease set from CASF-2016, and the ranker parameters are more important than the selector parameters for the accuracy of the method.

**FIGURE 2:**
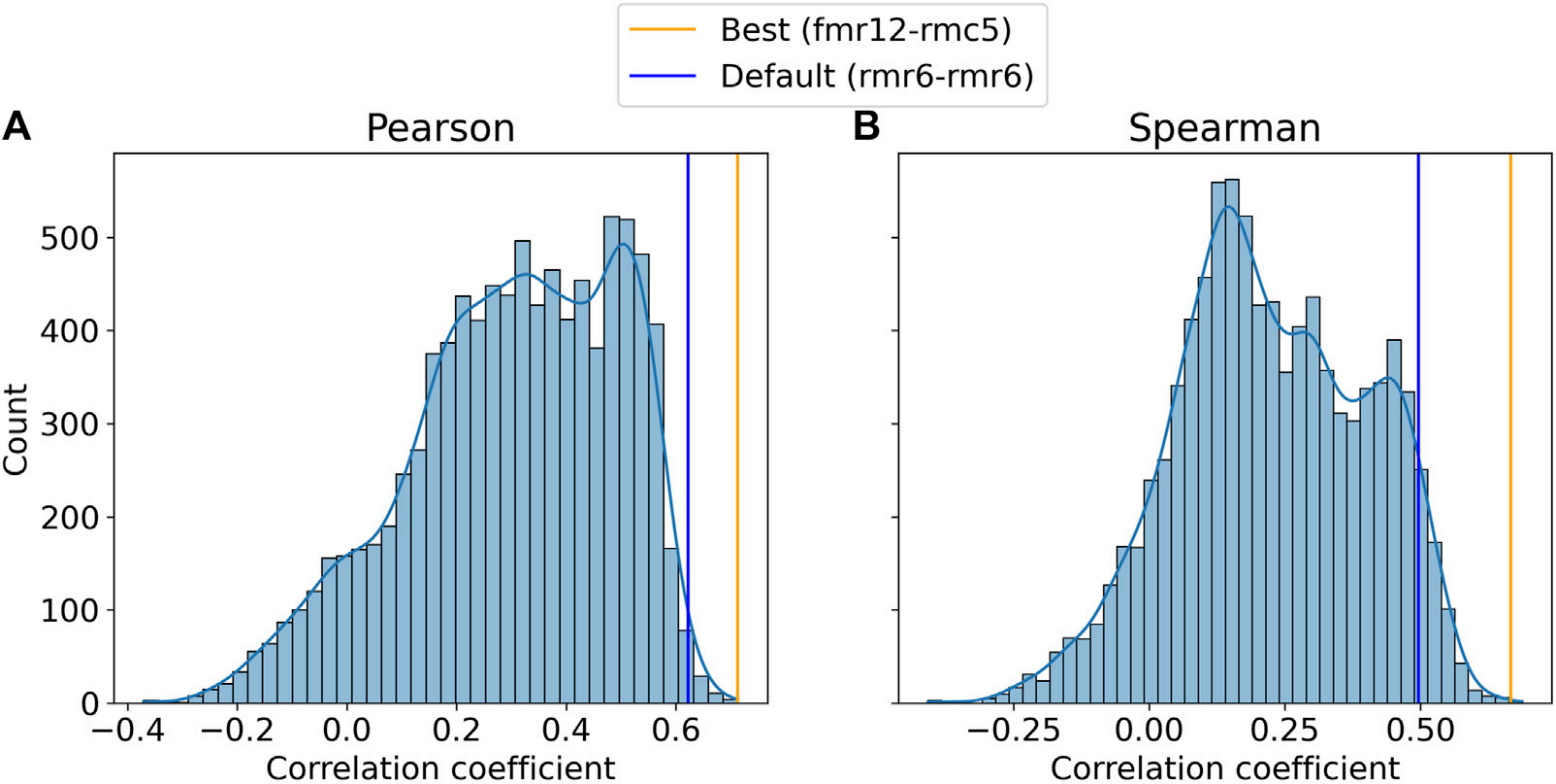
Distributions of Pearson **(A)** and Spearman **(B)** correlation coefficients between predicted and experimental binding affinities across all variations of the knowledge-based forcefield used by CANDOCK. The frequencies of the Pearson **(A)** and Spearman **(B)** correlation coefficients are plotted with the default and best performing parameter set coefficients denoted by blue and orange lines, respectively. The default parameter set (rmr6–rmr6) results in coefficients comparable to those resulting from the optimal parameter set (fmr12–rmc5) especially with regard to the Pearson correlation. These data support the use of the default and unbiased parameter set for the specific case of HIV-1 protease inhibitor binding prediction.

We analyzed our HIV-1 protease results by comparing the results of the default parameter set, rmr6–rmr6, to the best performing parameter set, fmr12–rmc5. Figure 2 shows that the best performing parameter set, fmr12–rmc5, yielded Pearson and Spearman correlations of 0.71 (*p*-value < 0.0001) and 0.67 (*p*-value < 0.0001), respectively, for the 30 HIV-1 protease–inhibitor complexes. The best performing parameter set marginally outperforms the default parameter set for CANDOCK, rmr6–rmr6, with Pearson and Spearman correlations of 0.62 (*p*-value < 0.001) and 0.50 (*p*-value < 0.01), respectively.

Both CANDOCK parameter sets were in the top 1% of Pearson scores, suggesting that the previously established default parameter set for assessing the ability of CANDOCK to accurately calculate the relative binding strength of HIV-1 protease–inhibitor complexes performs near optimally (Figure 2). This led us to continue further analysis with this unbiased parameter set rather than using the best one, thereby eliminating the risk of any overtraining.

### 3.2 Comparison to Other Docking Methods and Forcefields

**FIGURE 3:**
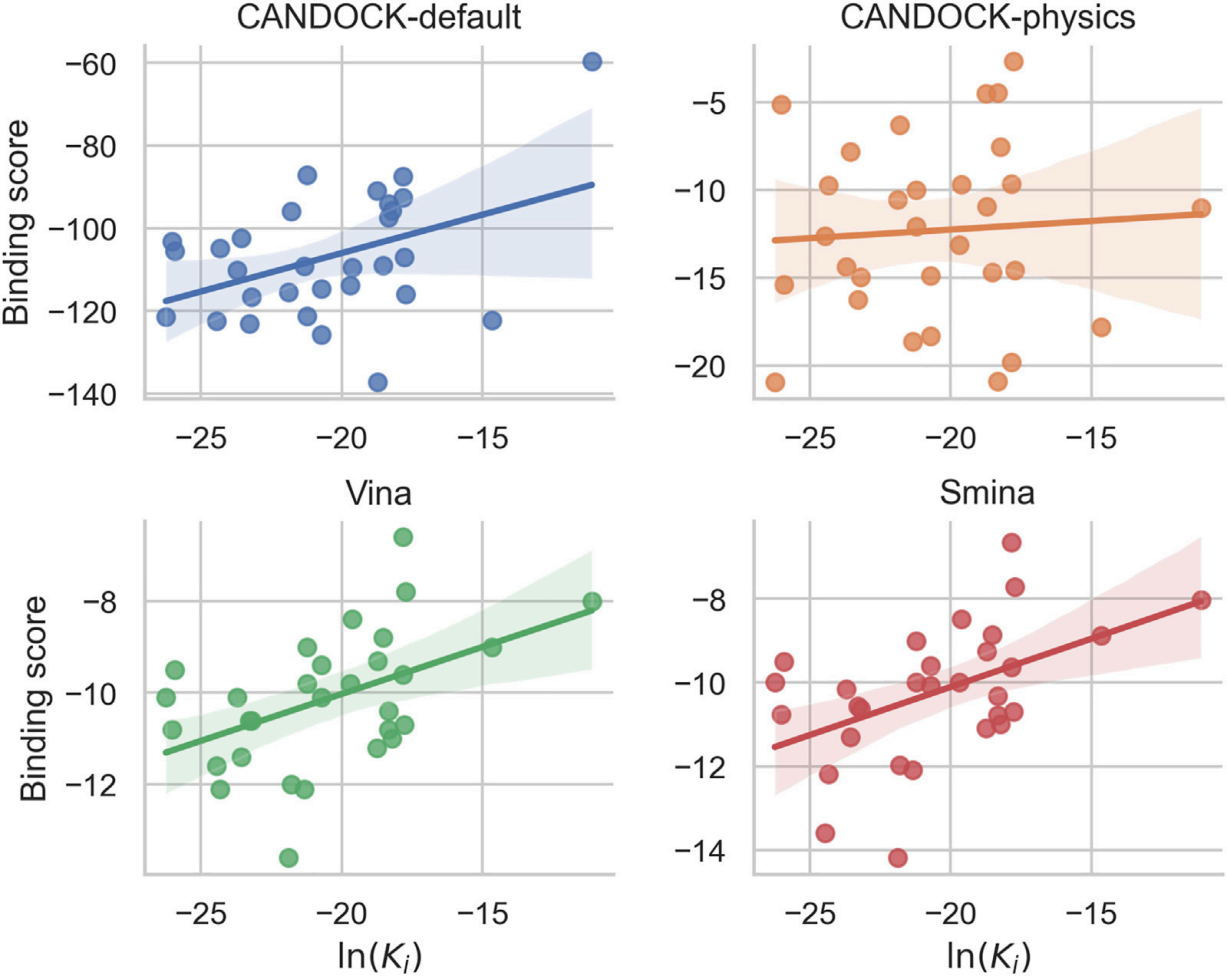
Comparison of predicted binding scores and known constants for 30 HIV-1 protease–inhibitor complex structures using four different docking protocols. Each panel plots the protein–ligand predicted binding scores from a particular docking protocol against the known binding constant [ln(K_*i*_)] for each HIV-1 protease–inhibitor complex with the linear regression line and the 95% confidence interval shaded. CANDOCK with the default parameters for the knowledge-based forcefield (blue) produced a Pearson correlation of 0.62 (*p*-value < 0.001), whereas CANDOCK with physics-based potential (orange), AutoDock Vina (green), and Smina (red) all had lower correlations of 0.07 (*p*-value = 0.7365), 0.48 (*p*-value = 0.0076), and 0.49 (*p*-value = 0.0061), respectively. These results illustrate the higher utility of CANDOCK with default parameters for predicting HIV-1 protease–inhibitor binding with respect to the other docking protocols and scoring functions.

We compared CANDOCK to two other well-established molecular docking protocols to determine their relative utility (Figure 3). For this comparison, we used protocols based on the Vina and Smina software (Morris et al., 2009; Trott and Olson, 2010). In addition, we also ran the predictions made by CANDOCK using a physics-based forcefield (CANDOCK-physics), GAFF, as a control (Wang et al., 2004a; Wang et al., 2006).

Figure 3 shows that Vina, which uses a scoring function involving knowledge-based and empirical components, generated Pearson and Spearman correlations of 0.48 and 0.50, respectively. Smina, a modified Vina with a custom scoring potential, performed similarly with correlations of 0.49 and 0.49 for Pearson and Spearman, respectively. Recall that the respective correlations were 0.62 and 0.50 for CANDOCK using the default KBF parameter set and 0.71 and 0.67 using the best performing one. Lastly, CANDOCK-physics was a control that resulted in 0.06 Pearson and 0.09 Spearman correlations. The physics-based potentials used to predict protein–ligand interactions showed little to no correlation to known binding affinities for the HIV-1 protease–inhibitor set.

These results combined indicate that CANDOCK-default is able to accurately predict the relative binding affinities with greater confidence than other docking protocols.

### 3.3 Discrimination of Active Versus Decoy Human Immunodeficiency Virus 1 Protease Inhibitors

We next assessed the discriminatory ability of CANDOCK to effectively identify active inhibitors of HIV-1 protease over decoys. We again compared the results of CANDOCK with default KBF parameters to Vina, Smina, and CANDOCK-physics. To accomplish this, we ran docking simulations using all four protocols on a set of active and decoy HIV-1 protease inhibitors from DUD-E (Mysinger et al., 2012).

**FIGURE 4:**
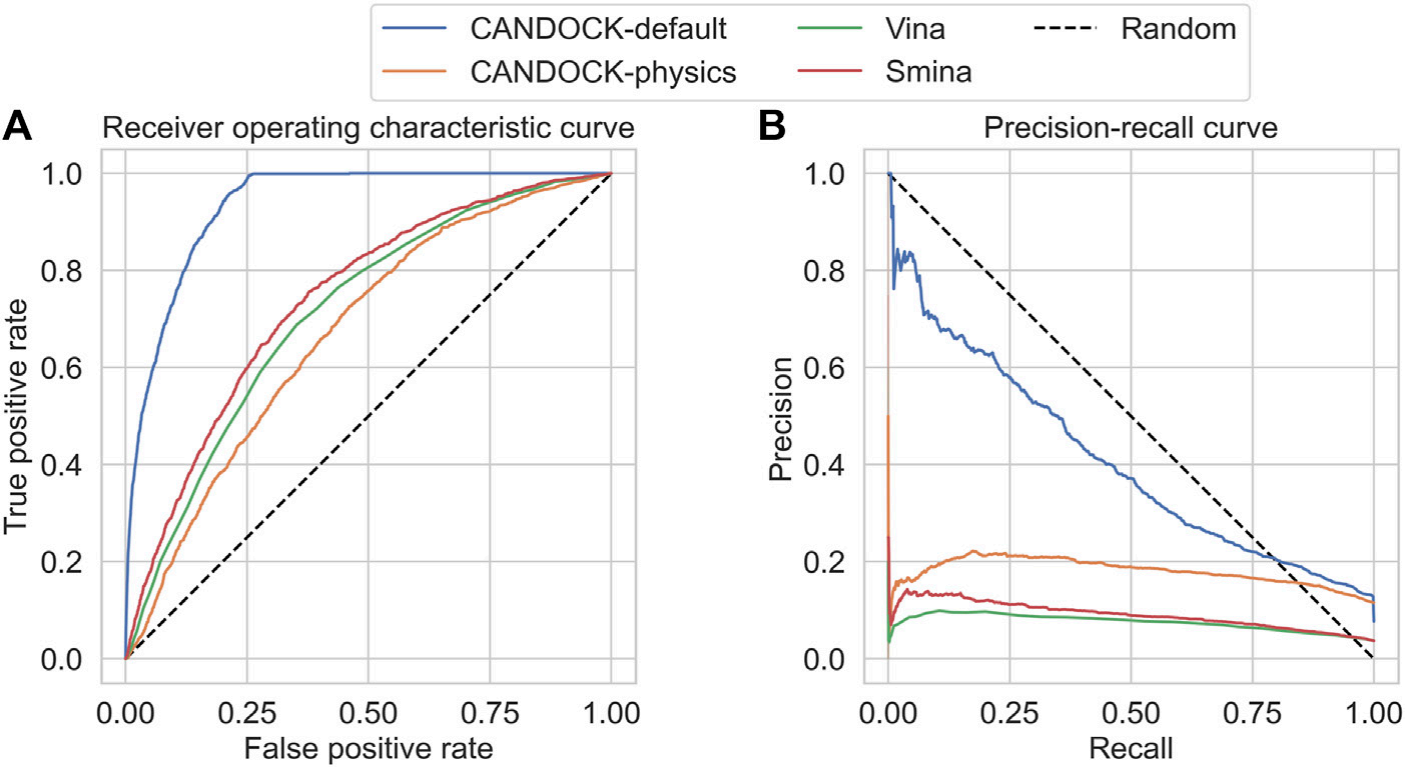
Receiver operating characteristic (ROC) and precision–recall (PR) curves for the HIV protease DUD-E set using four different docking protocols. The resulting area under the ROC (AUROC) curve values on the **(A)** are 0.94 for CANDOCK with the default KBF parameters (blue), 0.67 for CANDOCK with physics-based potential (orange), 0.71 for Vina (green), and 0.74 for Smina (red). The resulting area under the precision–recall curves (AUPRC) values on the **(B)** are 0.41 for CANDOCK-default, 0.18 for CANDOCK-physics, 0.08 for Vina, and 0.09 for Smina. These results provide further evidence that CANDOCK with its default KBF parameters outperforms the other docking protocols and scoring functions for prediction of binding affinity and activity of HIV-1 protease inhibitors.

For each protocol, the receiver operating characteristic (ROC) curve was generated based upon the sorted binding scores the protocol calculated for each active/decoy–protein pair; additionally, the AUROC was calculated for each protocol (Figure 4). The resulting AUROC values for all protocols were above 0.5, meaning they all performed better than random. CANDOCK with default KBF parameters performed the best with an AUROC of 0.94. The other three methods had much lower AUROC values with CANDOCK-physics at 0.67, Vina at 0.71, and Smina at 0.74. While all protocols performed well, CANDOCK-default displayed its superiority for binding score prediction.

We plotted the precision–recall curves for each protocol and calculated the area under these curves (AUPRC) to more thoroughly assess the protocols in discriminating active from decoy inhibitors (Figure 4). All four protocols scored under 0.5, which is common when there are imbalanced class sizes like in the DUD-E set. CANDOCK-default still outperformed the other three protocols with a AUPRC of 0.41. The remaining three protocols, CANDOCK-physics, Vina, and Smina, had AUPRCs of 0.18, 0.08, and 0.09, respectively.

These results show that in addition to CANDOCK predicting binding scores that correlate well with known binding affinities, the protocol is also capable of effectively choosing active inhibitors over decoys.

### 3.4 Discrimination of Known Active Versus Inactive Human Immunodeficiency Virus 1 Protease Inhibitors

To further investigate the efficacy of the CANDOCK protocol, we assessed its discrimination capability between known active HIV-1 protease inhibitors and known inactive compounds. This benchmark is similar to the DUD-E set; however, the inactive compounds are experimentally confirmed, as opposed to decoys that are generated based on chemical similarity to the active compounds. This provides a more robust test for CANDOCK to be compared to the other methods in their ability to identify HIV-1 protease inhibitors.

**FIGURE 5:**
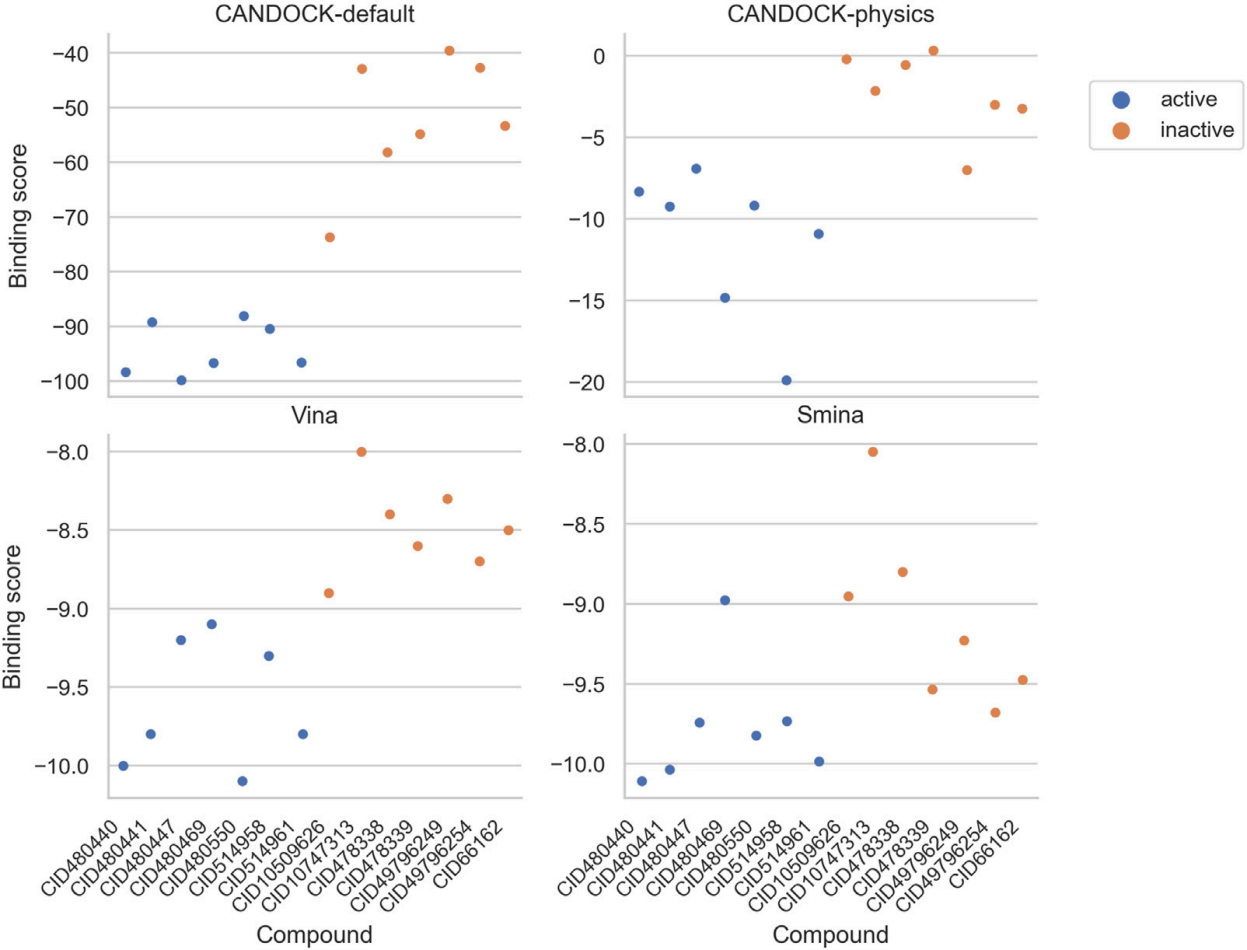
Predicted binding scores for HIV-1 protease inhibitors and inactive compounds using four different docking protocols. Each panel plots the protein–ligand predicted binding scores from a particular docking protocol for each of the seven active (blue) and seven inactive (orange) compounds. These results depict a distinct separation in binding scores for actives versus inactives using CANDOCK with default parameters and Vina.

For each protocol, we compared the resulting binding scores for each active and inactive compound (Figure 5) and visualized their separation based upon the strength of binding (active compounds should be in the lower left of the plot and the inactives should be in the upper right). CANDOCK-default and Vina distinguish the actives versus inactives clearly, while they group more closely for CANDOCK-physics and Smina.

**FIGURE 6:**
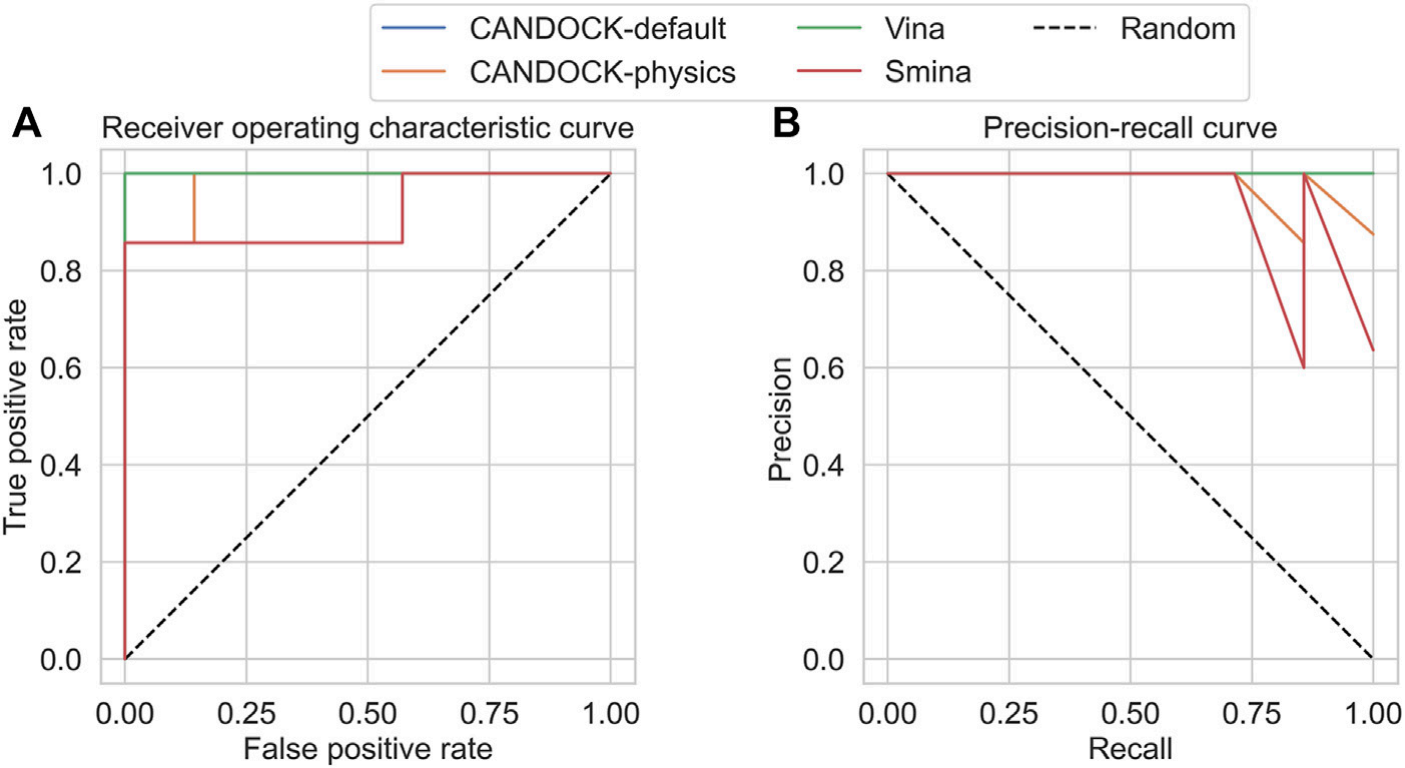
Receiver operating characteristic (ROC) and precision–recall (PR) curves for the HIV-1 protease actives and inactives (seven each). The resulting area under the ROC (AUROC) curve values on the **(A)** are 1.00 for CANDOCK with the default KBF parameters (blue), 0.98 for CANDOCK with physics-based potential (orange), 1.00 for Vina (green), and 0.92 for Smina (red). The resulting area under the precision–recall curves (AUPRC) values on the **(B)** are 1.00 for CANDOCK-default, 0.98 for CANDOCK-physics, 1.00 for Vina, and 0.95 for Smina. These results provide further evidence that CANDOCK with its default KBF parameters is an effective docking protocol and scoring function for prediction of binding affinity and activity of HIV-1 protease inhibitors.

To more explicitly determine the discriminatory ability, we subsequently generated the ROC curve based on the sorted binding scores the protocol calculated for each active/inactive–protein pair; additionally, the AUROC was calculated for each protocol (Figure 6). CANDOCK with default KBF parameters and Vina both performed the best with an AUROC of 1.00. The other two methods had lower AUROC values with CANDOCK-physics at 0.98 and Smina at 0.92. While all protocols performed well, CANDOCK-default and Vina displayed comparable efficacy for active versus inactive discrimination.

We plotted the precision–recall curves for each protocol and calculated the AUPRC to more thoroughly assess the protocols in discriminating actives from inactives (Figure 6). CANDOCK-default and Vina still outperformed the other two protocols with a AUPRC of 1.00, compared to AUPRC of 0.98 for CANDOCK-physics and 0.95 for Smina.

Overall, CANDOCK-default is effective at predicting active inhibitors for HIV-1 protease from a set of actives and inactives. Vina showed comparable results for this test, and both slightly outperformed Smina and CANDOCK-physics.

### 3.5 Comparison of Docking Simulation Times

**FIGURE 7:**
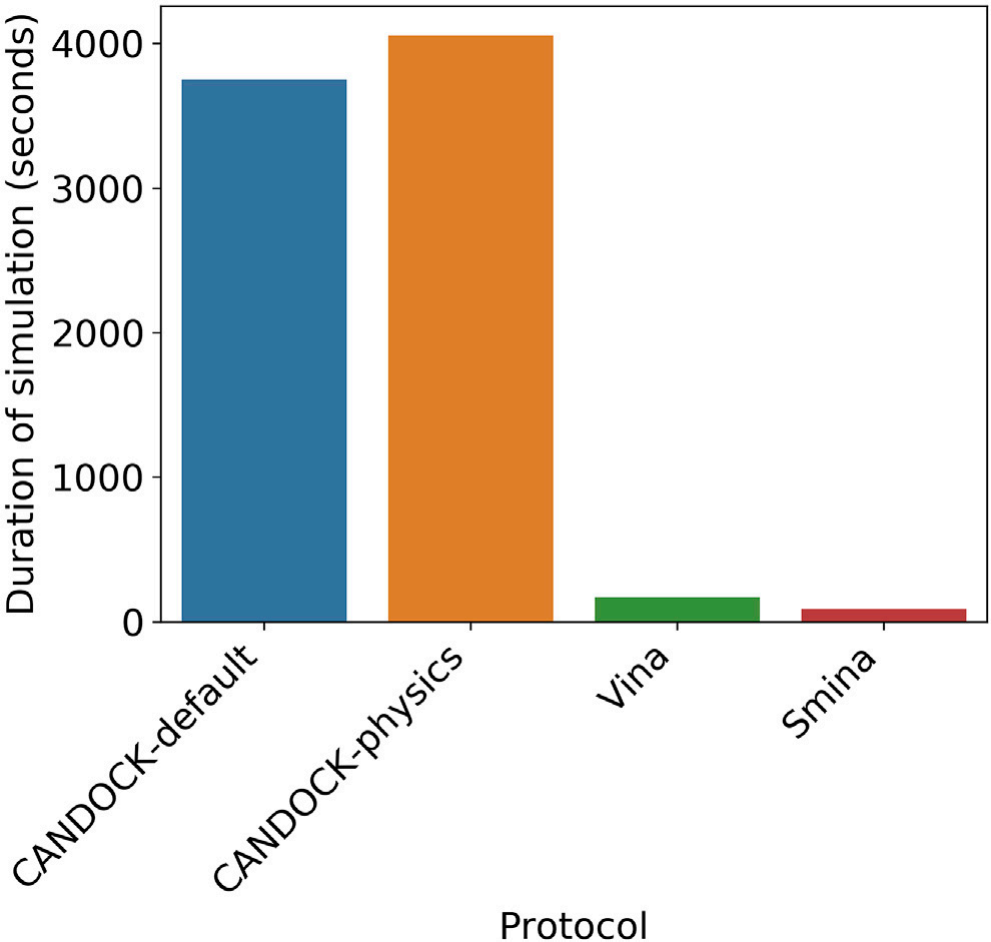
Average simulation time for four different docking protocols. The average times for the four protocols over 900 simulations are as follows: CANDOCK-default (blue) = 3,751 s, CANDOCK-physics (orange) = 4,055 s, Vina (green) = 169 s, and Smina (red) = 87 s. CANDOCK is slower than Vina and Smina, resulting in a tradeoff between accuracy and computational cost.

As another point of comparison for all four protocols, we assessed the average time it takes to generate the top pose for the HIV-1 protease–inhibitor complex. We ran the 30 protease–inhibitor complexes 30 times resulting in 900 simulations for each protocol. We then averaged the time of simulation, in seconds, for all 900 simulations and calculated the averages for each protocol (Figure 7). The results show that CANDOCK takes much longer to complete a single simulation, despite the scoring function with CANDOCK-default averaging 3,751 s and CANDOCK-physics averaging 4,055 s. Vina and Smina are comparable to each other and much faster than CANDOCK, with an average time for simulation of 169 and 87 s, respectively.

Balancing the computational cost with the accuracy of the protocol is important to consider, especially if the compound sets being tested are very large (>1,000) or the available computing power is limited.

### 3.6 Limitations and Future Work

The size of the set of HIV-1 protease–inhibitor complexes used for the parameterization and correlation calculations is limited to only 30 members. A larger set would enable better parameterization and assessment of the protocols by providing more variability in the chemical space of the ligands. Moreover, a larger set of known active and inactive compounds would allow for a much more rigorous comparison of these methods in their ability to discriminate active inhibitors from inactive compounds.

The datasets used herein all focused on the wildtype HIV-1 protease, but the prevalence of mutations that confer drug resistance is of great concern in HIV treatment. Future work will assess the sensitivity of CANDOCK and the other protocols to inhibitors of HIV-1 protease mutations. Since CANDOCK has already been shown to be effective in predicting and designing specific inhibitors, demonstrating the efficacy of the CANDOCK protocol in the prediction of binding affinities to any protease mutant will greatly aid in future drug design efforts for effective, broad-spectrum protease inhibitors to replace the cocktails that are currently used (Larocque et al., 2017; Ma et al., 2017). Moreover, if our results were generalizable to predict accurate binding between HIV-1 protease mutants and their inhibitors, it would allow for a precision medicine approach to protease inhibitor efficacy prediction (Jenwitheesuk and Samudrala, 2003; Jenwitheesuk et al., 2004; Wang et al., 2004b; Jenwitheesuk and Samudrala, 2005a;2005b; Jenwitheesuk et al., 2005; Jenwitheesuk and Samudrala, 2007; Jenwitheesuk et al., 2008).

Leveraging some of our recently developed programs, based on machine learning and graph neural networks, we can iteratively select synthetically feasible bioactive protease inhibitors based on bioactivity data and CANDOCK-generated pose of molecules (Majumder et al., 2018; Wijewardhane et al., 2020). Exploration of CANDOCK efficacy on other HIV-1 targets, such as reverse transcriptase, would enable proteomic-based drug discovery, which we have shown to be useful for drug repurposing, and could lead to more potent HIV-1 therapeutics (Chopra et al., 2016; Chopra and Samudrala, 2016; Hernandez-Perez et al., 2017; Majumder et al., 2017; Fine et al., 2019; Mangione et al., 2020; Robertson et al., 2020).

## 4 Conclusion

We evaluated four different docking protocols for their effectiveness in predicting HIV-1 protease–inhibitor binding affinities. We assessed these protocols by correlation to known binding affinities and by ability to discriminate between known active and decoy inhibitors. The results from both these computational experiments showed that the CANDOCK protocol with its all-atom knowledge-based forcefield was superior to these other protocols.

Overall, we show that CANDOCK accurately predicts the relative binding affinities for HIV-1 protease inhibitors when compared to and greatly outperforms popular publicly available molecular docking protocols. The efficacy demonstrated by CANDOCK in our study indicates that it will be very useful for the repurposing, discovery, and design of novel HIV-1 protease inhibitors.

## Data Availability

The data generated for this study can be found at http://compbio.buffalo.edu/data/in_hiv_candock.
